# Next-generation direct reprogramming

**DOI:** 10.3389/fcell.2024.1343106

**Published:** 2024-02-02

**Authors:** Riya Keshri, Damien Detraux, Ashish Phal, Clara McCurdy, Samriddhi Jhajharia, Tung Ching Chan, Julie Mathieu, Hannele Ruohola-Baker

**Affiliations:** ^1^ Department of Biochemistry, School of Medicine, University of Washington, Seattle, WA, United States; ^2^ Institute for Stem Cell and Regenerative Medicine, School of Medicine, University of Washington, Seattle, WA, United States; ^3^ Department of Bioengineering, College of Engineering, University of Washington, Seattle, WA, United States; ^4^ Institute for Protein Design, University of Washington, Seattle, WA, United States; ^5^ Department of Comparative Medicine, School of Medicine, University of Washington, Seattle, WA, United States

**Keywords:** transdifferentiation, direct reprogramming, partial reprogramming, pioneer factors, aging, signaling, skeletal muscle, cardiac muscles

## Abstract

Tissue repair is significantly compromised in the aging human body resulting in critical disease conditions (such as myocardial infarction or Alzheimer’s disease) and imposing a tremendous burden on global health. Reprogramming approaches (partial or direct reprogramming) are considered fruitful in addressing this unmet medical need. However, the efficacy, cellular maturity and specific targeting are still major challenges of direct reprogramming. Here we describe novel approaches in direct reprogramming that address these challenges. Extracellular signaling pathways (Receptor tyrosine kinases, RTK and Receptor Serine/Theronine Kinase, RSTK) and epigenetic marks remain central in rewiring the cellular program to determine the cell fate. We propose that modern protein design technologies (AI-designed minibinders regulating RTKs/RSTK, epigenetic enzymes, or pioneer factors) have potential to solve the aforementioned challenges. An efficient transdifferentiation/direct reprogramming may in the future provide molecular strategies to collectively reduce aging, fibrosis, and degenerative diseases.

## 1 Introduction

While the concept that mature cell states are stable holds the key for homeostasis of an organism, the long-held believe was that this state cannot ever be reversed. This fallacy has gradually broken down, most notably by 2012 Nobel-winning studies by John Gurdon and Shinya Yamanaka. In 1962 Prof. Gurdon showed that in specific conditions, the nucleus of a differentiated cell is capable of de-differentiating into the pluripotent stage. This revolutionary finding changed modern biology and marks the initiation of reprogramming field. Later studies revealed the reversion of epigenetic marks as the key mechanism of the process. Gurdon’s nuclear transfer work led to animal cloning from tadpoles to Dolly the sheep, to genetically modified pigs and cows, etc. Yamanaka’s work then identified the molecules capable of reprogramming adult cells into a pluripotent stage (induced pluripotent stem cells, iPSC) (Takahashi and Yamanaka, 2006; Takahashi et al., 2007; Takahashi and Yamanaka, 2016). The Yamanaka factors (OCT3/4, SOX2, KLF4, c-MyC) are now widely used not only for reprogramming but also for partial reprogramming that leads to rejuvenation of tissues (Ocampo et al., 2016; Browder et al., 2022).

Yet another kind of reprogramming was emerging from the basic science field, now dubbed direct reprogramming, or transdifferentiation (we use the terms interchangeably from here on). During transdifferentiation a differentiated cell changes its fate to another, more desired differentiated cell type, without entering a pluripotent stage. The first identified transcription factor capable of directly reprogramming fibroblasts to skeletal muscles was MyoD (Davis et al., 1987; Weintraub et al., 1989). Many other lineage-specific transcription factors capable of transdifferentiating a target cell have since been identified (Wang et al., 2021).

While transdifferentiation can be induced on demand using transcription factor overexpression, some endogenous transdifferentiation have been observed *in vivo* as well; from jellyfish to mammals, transdifferentiation has shown to play an important role in normal animal adaptation. Some of the naturally occurring transdifferentiation processes are: immune cell transdifferentiation in vertebrates and *Drosophila melanogaster* (Csordás et al., 2021), alpha to beta-cell transdifferentiation in pancreatic islets (Thorel et al., 2010) and the white adipocyte transdifferentiation to brown adipocytes due to cold induced transcriptional and epigenetic modifications (Roh et al., 2018).

Whether induced or endogenous process, in general, a pioneer factors (PF) act as the first responders in direct reprogramming by binding and opening closed chromatin. Transdifferentiation epigenetic factors are then recruited, a necessary step for maintaining the open chromatin state to activate transcription of key lineage-specifying genes. In general, pioneer factors have the capacity to bind a multitude of chromatin locations and thereby in a regulated manner affect an entire suite of genes destined to activate a particular cell fate in a regulated manner. Recent data suggest that pioneer factors may be more general than we have previously appreciated (Wang et al., 2022a). Hence today, it is not clear if each transdifferentiation lineage is regulated by a specific pioneer factor, or if a universal PF for transdifferentiation (capable of initiating multitude of direct lineage reversions) is still to be identified.

In general, transdifferentiation studies have unveiled the opportunities and offer applications in regenerative therapies, such as cell replacement therapy or immunotherapy (Wang et al., 2021). The key question, and the topic of this review is to identify new, feasible methods to induce specific, high efficiency and targeted transdifferentiation. These newest technologies may lead to the next-generation direct reprogramming that due to increased accuracy and efficiency may become invaluable for disease modeling, and for future therapeutics.

## 2 Pioneer factors in transdifferentiation

**FIGURE 1:**
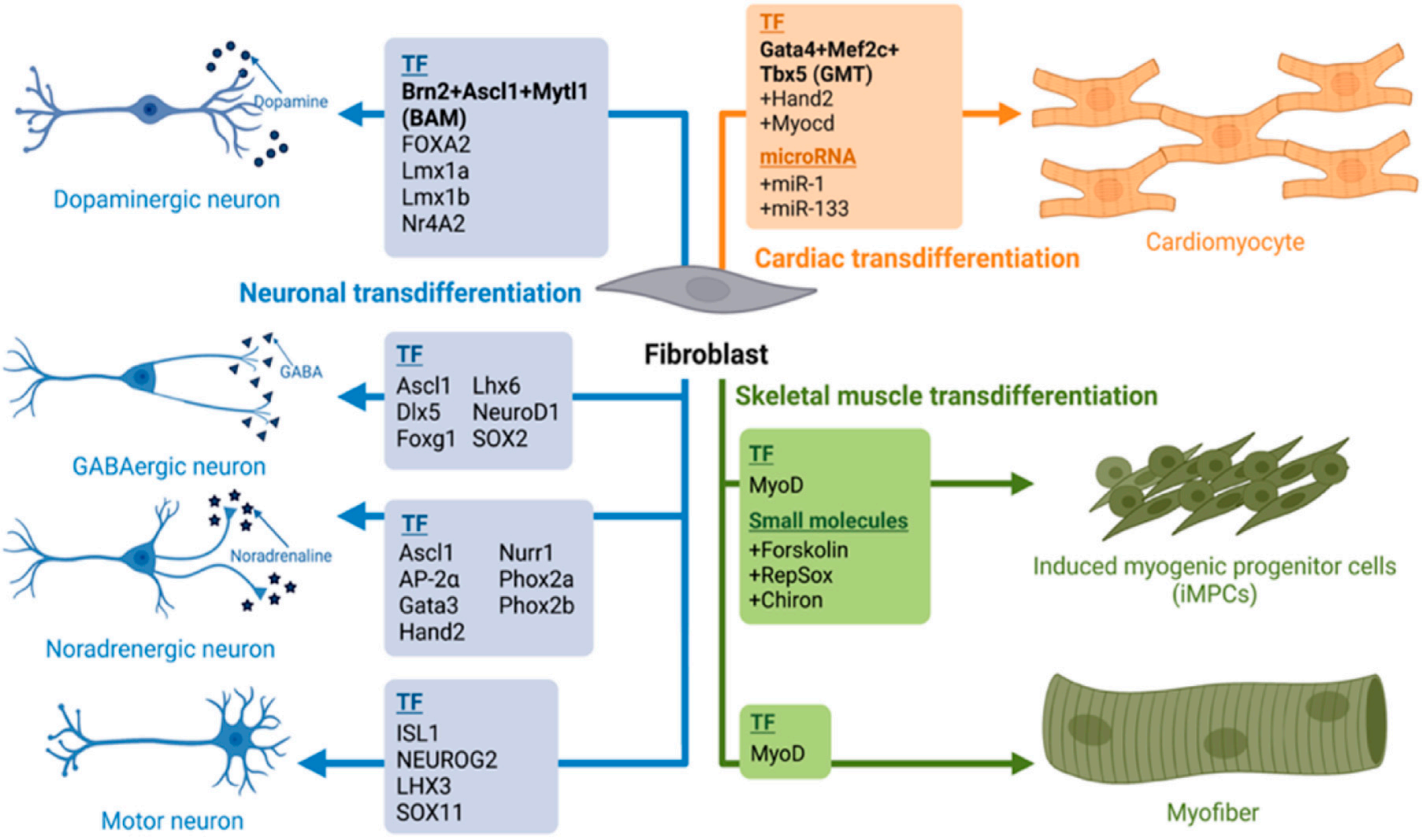
A summary of transdifferentiation fibroblast into skeletal, cardiac muscles and neurons. We summarize here various cocktails of transcription factors and their combination with small molecules or micoRNAs their lineage specific trans differentiation such as Dopaminergic neurons (Vierbuchen et al., 2010; Pfisterer et al., 2011a; Pfisterer et al., 2011b), GABAminergic (Lee et al., 2020; Bruzelius et al., 2021); Noradregenic neuron (Ainsworth et al., 2018); Myofibre (Davis et al., 1987; Tapscott et al., 1988; Weintraub et al., 1991); iMPCs (Qabrati et al., 2023) and Cardiomyocyte (Ieda et al., 2010; Qian et al., 2012; Song et al., 2012; Wada et al., 2013). Image created by Biorender.

An exogenous expression of master regulators, lineage specific transcription factors (TF) has been one of the most reliable methods of transdifferentiation. For example, direct reprogramming of mouse fibroblast to myoblast was originally achieved by the exogenous expression of MyoD (Davis et al., 1987; Tapscott et al., 1988; Weintraub et al., 1991). Furthermore, a fusion of transcriptional activation domain VP-64 to N-terminus of wild-type MyoD increased the transdifferentiation efficiency in human fibroblasts (Kabadi et al., 2015). A combination of three transcription factors Gata4, Mef2c, and Tbx5 (GMT) induced transdifferentiation of mouse fibroblasts into functional cardiomyocytes *in vitro* and *in vivo* upon transplantation (Ieda et al., 2010; Qian et al., 2012; Song et al., 2012). For human fibroblasts, addition of MESP1 and MYOCD to the GMT cocktail was required for the cardiomyocyte conversion (Wada et al., 2013). The first direct neuronal reprogramming (mouse astrocytes to neurons, iN) was induced with the expression of either Pax6, Neurog2 or Ascl1 (Heins et al., 2002; Berninger et al., 2007). To convert both mouse and human fibroblasts into iNs required the use of three TFs: Brn2, Ascl1 and Mytl1 (BAM) (Vierbuchen et al., 2010; Pfisterer et al., 2011a; Pfisterer et al., 2011b). In all of the examples above, the key transcription factors acted by binding and opening the chromatin of the critical genes and were coined pioneer factors (PF) due to this capacity. Among the neural transdifferentiation, Ascl1 was shown to act as a pioneer factor, binding and opening chromatin of neural genes, allowing their expression (Wapinski et al., 2017; Wang et al., 2022a), while Brn2 and Mtl1 were used as secondary TFs binding to the newly opened regions (Vierbuchen et al., 2010; Ambasudhan et al., 2011; Mall and Wernig, 2017; Wapinski et al., 2017). Presently, a multitude of pioneer factors with their companioning TFs have been identified in transdifferentiation processes (Figure 1). However, despite of the master transcription factor overexpression the reprogramming efficiency has not been sufficient for efficient chronic disease modeling, or clinical trials. Eventually, epigenetic abnormalities, metabolic maturation, aging and inflammatory response are some of the major roadblocks of direct reprogramming today that need to be addressed.

## 3 Targeting signaling pathways augments transdifferentiation

**FIGURE 2:**
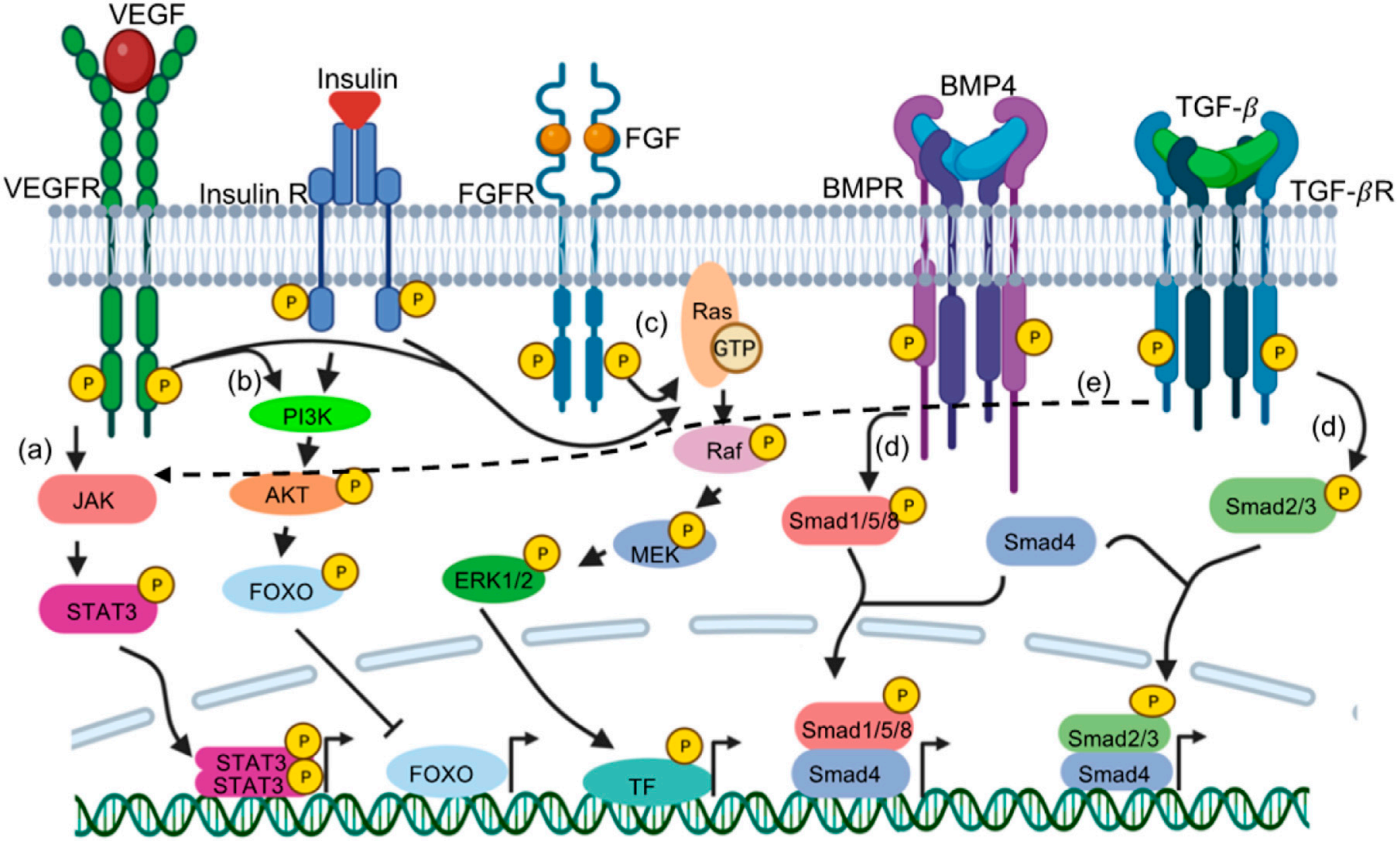
Signaling cascades augments transdifferentiation through gene expression. Various receptor tyrosine kinases (RTKs) or receptor Serine/Threonine Kinase (RSTKs) have been targeted in various direct reprogramming. RTKs such as VEGFR, FGFR, Insulin receptors acts through **(A)** JAK-STAT signalling, activated Janus Kinase downstream of the receptor phosphorylates STAT3. STAT3 dimers dependent gene expression is initiated. **(C)** MAPK pathway- RasGTP results in further phosphorylation of Raf, MEK and ERK1/2; phosphorylated ERK1/2 phosphorylate transcription factors (TF such as JUN,FOS, MYC, etc.) that activates their target genes. **(B)** PI3K pathway Activated PI3K downstream of the receptor phphorylates AKT. pAKT phosphorylation repress transcription factors such as FOXO (shown as example), BAD, TSC2, p27 while activates other transcription factors such as MDM2 and eNOS. RSTKs such as BMPR and TGF- β R phosphorylates **(D)** R-Smads: Smad1/Smad5/Smad8 and Smad2/Smad3 respectively. These phosphorylated R-Smads forms complex with co-Smad4 that translocate in the nucleus resulting in transactivation of target genes. **(E)** TGF- β R can also acts through JAK-STAT pathway (shown by dashed arrow). Image created by Biorender.

Modulating signaling pathways has been another strategy for enhancing efficiency during transdifferentiation. Co-culturing mouse primary fibroblasts from various tissue origins with the C2C12 myoblasts in differentiation media, induced myogenic reprogramming in the fibroblasts (Iberite et al., 2022). This emphasized the importance of extrinsic signaling in cellular reprogramming. Inhibition of pathways such as TGF-β/activin pathway, BMP4 pathway, TNF-α and activation of canonical WNT signaling, IGF signalling, Follistatin pathway, GDF11 signaling, hGH pathway have shown to improve skeletal muscles trans differentiation (Boularaoui et al., 2018). Specifically, MyoD-induced myogenic transdifferentiation can be enhanced by IGF activation or BMP4 inhibition (Cacchiarelli et al., 2018). MyoD-E47 heterodimers can be inhibited by Id proteins that are downstream of BMP4 activation (Lassar et al., 1991). It is therefore plausible that BMP4 inhibition is beneficial for skeletal transdifferentiation via induced MyoD activity. Further, activation or inhibition of pathways such as FGF, WNT, NOTCH and IGF have increased the efficiency of cardiac transdifferentiation, as well as the maturity. BMPR/TGFbR inhibition has shown to be critical for guiding fibroblasts to the iN fate (Ladewig et al., 2012; Lu et al., 2013; Mertens et al., 2015; Pfisterer et al., 2016; Kim et al., 2018; Yang et al., 2019; Liu and Wang, 2020; Mall and Wernig, 2017; Bruzelius et al., 2021). Increasing activity of Wnt is also one of the most common strategies to improve the direct conversion to iNs. Indeed, Wnt activation, through the inhibition of GSK3β (CHIR99021) promotes iN generation (Dai et al., 2015; Mertens et al., 2015; Mall and Wernig, 2017; Kim et al., 2018; Bruzelius et al., 2021), presumably through activating neuron specific transcription factors (such as Ngn1, NeuroD and Brn3a). In addition to BMPR/TGFbR inhibition and Wnt activation, activity of the sonic hedgehog (SHH) pathway is also critical in neuron specification both from iPSC and from fibroblasts through direct conversion (Kriks et al., 2011; Arenas et al., 2015; Qin et al., 2020). Small molecules targeting specific signaling pathways can promote successful cardiac transdifferentiation through FGFR and VEGFR activation (Muraoka et al., 2019), or inhibition of TGF - β R and NOTCH (Ifkovits et al., 2014; Abad et al., 2017; Mohamed et al., 2017). These studies in general demonstrate the potential of using an outside-in approach of activating transcriptional programs for direct reprogramming (Figures 2A–E). However, more potent and specific regulators for receptor signaling are required to increase the efficiency.

## 4 Correct epigenetic modifications are critical in transdifferentiation

Direct reprogramming requires massive, but precise epigenomic alteration to ensure a proper cell fate change. Repatterning of H3K27me3, H3K4me3 and DNA methylation marks during fibroblast to cardiomyocyte direct reprogramming is one such example (Liu et al., 2016). The pioneer transcription factors such as MyoD, Ascl1 or GATA4 regulate the chromatin landscape through interaction with epigenetic modifiers (Molkentin et al., 1995). For example, MyoD as a pioneer factor, through epigenetic alterations allows access of chromatin to other transcription cofactors such as MEF2 proteins, Sp1, Pbx and Six proteins, that bind to flanking sequences of the E-motifs for synergistic transactivation, presumably through epigenetic alterations (Yun and Wold, 1996; Biesiada et al., 1999; Guo et al., 2003; Berkes et al., 2004; Liu et al., 2010; Taylor and Hughes, 2017). Studies have shown that modulation of epigenetic proteins can significantly improve the efficiency of reprogramming (Dal-Pra et al., 2017). For example, inhibition of Ezh2, the histone methyltransferase of PRC2, improves cardiac reprogramming (Hirai and Kikyo, 2014) and ablation of KMT2B, a histone methyltransferase, drastically reduces neuron reprogramming (Barbagiovanni et al., 2018).

The acquired DNA methylation pattern also correlates to the age of the donor (epigenetic aging-clock), representing an advantage of transdifferentiated neurons over iPSC-derived neurons for the study of age-dependent pathologies (Mertens et al., 2021). While this is an advantage in this case, it is also a barrier to efficient conversion as old fibroblasts conserve the hypermethylation pattern (Zhang et al., 2016). Various epigenetic modifiers have been utilized to improve transdiffererntiation (Table 1). For example, small molecules such as HDACs (Histone deacetylase) inhibitor trichostatin A (Fernandes et al., 2023), valproic acid (Hu et al., 2015; Qin et al., 2020) or the histone demethylase inhibitor parnate (Liu and Wang, 2020) have been used in the neuron conversion cocktails. Interestingly, the addition of ascorbic acid to the conversion media is also critical as it can activate both TET and Jumonji-C domain-containing histone demethylases leading to a more efficient epigenome rewiring (Lu et al., 2013; Lee Chong et al., 2019; Qin et al., 2020).

**TABLE 1:** Role of epigenetic modifiers in transdifferentiation.

Factors/Conditions	Epigenetic modification	Effect	References
GSK126	Ezh2 (H3K27 methyltransferase) inhibition	Improved cardiac reprogramming	Hirai and Kikyo (2014)
UNC0638	G9a and GLP (H3K9 methyltransferases) inhibition	Improved cardiac reprogramming	Hirai and Kikyo (2014)
*Kmt2b−/−*	KMT2B (H3K4 methyltransferase) ablation	Improved neuronal reprogramming	Barbagiovanni et al. (2018)
trichostatin A	HDACs (Histone deacetylase) inhibition	Improved neuronal reprogramming	Fernandes et al. (2023)
valproic acid	HDACs (Histone deacetylase) inhibition	Improved neuronal reprogramming	Qin et al. (2020), Hu et al. (2015)
ascorbic acid	histone methyltransferase inhibitors	Improved neuronal reprogramming	Lu et al. (2013), Qin et al. (2020)
Parnate	histone demethylase inhibition	Improved neuronal reprogramming	Liu and Wang (2020)
Tet3 overexpression	DNA demethylase overexpression	Improved neuronal reprogramming	Zhang et al. (2016)
BIX01294	G9a and GLP (H3K9 methyltransferases) inhibition	Improved cardiac reprogramming	Cao et al. (2016)
AS8351	KDM5B histone demethylase inhibition	Improved cardiac reprogramming	Cao et al. (2016)

Further, non-coding RNAs have been utilized to enhance transdifferentiation efficiency. A cocktail of microRNAs including miR-1, miR-133, miR-208 and miR-499 was found to transdifferentiate mouse embryonic fibroblasts into cardiomyocyte-like cells (Jayawardena et al., 2012; Paoletti et al., 2020). Since then, studies have shown that different combinations of microRNAs and transcription factors can reprogram human fibroblasts with variable efficiencies (Nam et al., 2013; Muraoka et al., 2014). Some long noncoding RNAs such LncMyoD or Linc-RNA activator of myogenesis (Linc-RAM) and steroid receptor RNA activator (SRA) are known to promote myogenic transdifferentiation through modulating chromatin accessibility of several transcription factors (Yu et al., 2017; Dong et al., 2020).

## 5 Metabolic changes in transdifferentiation

While metabolic maturation has been a bottleneck in iPSC-based differentiation in general, the trans-differentiation process is shown (at least in the case of cardiomyocytes) to maintain the cellular metabolism observed in adult cells (Mathieu and Ruohola-Baker, 2017; Zhou et al., 2017; Miklas et al., 2019; Jahng et al., 2022). However, while both cell types (fibroblasts and transdifferentiated cells) are cells with mature metabolism, hence capable of utilizing a multitude of substrates for cellular energy, the demand for energy production in muscle is notably larger than in fibroblasts. Hence the number of mitochondria and their respiration capacity is expected to increase during direct reprogramming from fibroblasts to muscle cells. Accordingly, MyoD is shown to regulate skeletal muscle oxidative metabolism (Shintaku et al., 2016). Similarly single-cell RNA sequencing of GMT-induced transdifferentiation of fibroblasts to cardiomyocyte revealed dramatic metabolic alteration, and inhibition of oxidative metabolism impaired cardiomyocyte transdifferentiation (Zhou et al., 2017; Zhou et al., 2019; Jahng et al., 2022). A metabolic remodeling is also observed in neuronal transdifferentiation (Zheng et al., 2016). A mitochondrial mass and activity increase is driven by PGC-1α and ERRγ, two master regulators (Zheng et al., 2016). In accordance, an overexpression of the neuron-enriched mitochondrial proteins further boosted the conversion efficiency (Russo et al., 2021).

Interestingly metabolic changes due to aging or pathological conditions may transmit to the direct-reprogrammed cell types. This presents an opportunity to utilize transdifferentiated cells for disease modeling and search for therapies. For example, in Parkinson disorder, an impaired mitophagy (with parkin mutations) leading to metabolic stress is correlated to loss of neurons (Schwartzentruber et al., 2020). In Huntington’s disease, the increased ROS (reactive oxygen species) levels lead to DNA damage and neuronal loss (Victor et al., 2018). Fibroblast derived from an aged donor showed reduced mitochondrial activity and increased ROS levels (Huh et al., 2016; Kim et al., 2018), and neurons transdifferentiated from aged fibroblasts display reduced mitochondrial activity as well as reduced transdifferentiation efficiency (Kim et al., 2018). The efficiency of aged fibroblast conversion to neurons can be partially rescued by co-expression of either of an anti-apoptotic or antioxidant genes like BCL2 or SOD1 (Gascón et al., 2016; Russo et al., 2021). Furthermore, transdifferentiated adult cells may allow further dissection of the interdependence of metabolic, epigenetic and morphological maturation. For example, one could in the future test if the proteostasis index in these cells affects their health, regardless of their aging state.

## 6 Next-generation approaches in transdifferentiation

While above-described advances in Pioneer factor-, Signaling pathway-, Epigenetic- and Metabolic-regulation of the process have pushed the direct reprogramming approaches forward, major challenges still exist. Aging, inflammatory responses, fibrosis, metabolism and epigenetic barriers are the real, remaining challenges of direct reprogramming. Utilizing modern cutting edge technologies (including Artificial Intelligence (AI) based protein design) we can overcome some of these barriers. We discuss below some of the top-notch existing methods that have been less explored, and propose some novel methods of direct reprogramming that may revolutionize the personalized regenerative medicine.

### 6.1 Modulating signaling pathways using novel designed minibinders

Targeting signaling pathways with stable, ultra-specific and high affinity agonists and antagonists can be a monumental step forward in transdifferentiation. Recent advances in protein design now allow development of such powerful tools. Novel AI-based protein prediction and protein design (AlphaFold 2, RoseTTAFold, RFDiffusion) approaches are promising towards understanding disease, with a broad range of applications (Baek et al., 2021; Jumper et al., 2021; Bordin et al., 2023; Cheng et al., 2023; Watson et al., 2023). Since the cellular fate in direct reprogramming can be controlled by outside-in signaling (see Section 3), designed, highly specific and potent minibinder antagonists or agonists can potentially modulate the cellular fate to desired state. Synthetic agonists for TRKA receptor, EGFR, Tie2 receptor, FGFR1/2c, TGFbR or WNT (Janda et al., 2017; Zhao et al., 2021; Cao et al., 2022) allow precise analysis of specific splice variants and isomers of the receptors in multiple context, including transdifferentiation. These minibinders are precisely designed to target specific receptors and can also distinguish the receptor isoforms with minimal side effects which is not achievable with the natural ligands (Ocampo et al., 2016; Janda et al., 2017; Edman et al., 2023). An additional advantage of designed proteins is their greater stability (better half-lives) to ensure a sustained signaling response (Edman et al., 2023). One such example is usage of designed protein FGFR1/2c-isoform antagonist or agonist during pluripotent stem cell to endothelial cell differentiation. In the presence of FGFR1/2c designed miniprotein antagonist the pluripotent cells shift the fate completely to pericytes state instead of endothelial and in the presence of agonist iENDOs (iPSC-derived endothelial cells) shift to arterial vascular fate (Edman et al., 2023). These designed RTK agonists or antagonists can be utilized either in combination with transcription factors/pioneer factors, or plausibly in the future individually to reprogram the fibroblasts into desired tissues.

Designed minibinders may also be used to alleviate the problem of inflammation and fibrosis. Both inflammation and fibrosis have shown to be problematic for reprogramming. For instance, adult or neonatal fibroblast with increased inflammatory and fibrotic response show low efficiency of direct cardiac reprogramming (Chen et al., 2012; Zhao et al., 2015). Inhibiting the inflammatory chemokines or cytokines might improve the efficiency (Muraoka et al., 2019). Further, myocardial infarction triggers an inflammatory response in the myocardium that results in cardiac fibroblast activation. Recent advances in designing minibinders for IL (Interleukin)-receptors (Cao et al., 2022) may give a path for a new approach for reducing inflammation towards more robust direct reprogramming.

### 6.2 Direct reprogramming using mRNA

DNA delivery or viral delivery of transcription factors or microRNA faces clinical obstacles due to potential tumorigenic potential and immunogenic response. Injecting the mRNA or synthetic modified mRNA (modRNA; RNA with pseudoUridine, promises to offer a safer approach with potentially lower inflammation and immunogenicity and reduced risk of genomic integration (Karikó et al., 2008; Chien et al., 2014). For example, injecting synthetic VEGF-A modified mRNA (modRNA) can lead to efficient expansion and differentiation of cardiac progenitor cells (Zangi et al., 2013). For direct reprogramming of non-cardiomyocytes into cardiomyocytes modRNA has been used to express multiple transcription factors (Gata4, Mef2c, Tbx5, Hand2) and helper proteins [dominant-negative (DN)-TGFβ, DN-Wnt8a, and acid ceramidase] (Kaur et al., 2021). Further, transfection of MyoD-mRNA can reprogram MEFs into induced myogenic progenitor cell fate (iMPC) (Qabrati et al., 2023). These transgene free derived iMPCs successfully engrafted and restored dystrophin expression in DMD (Duchenne muscular dystrophy) mice (Qabrati et al., 2023). While exciting, further advances in efficiency and specificity of mRNA or modRNA delivery is required for therapeutic purposes.

### 6.3 CRISPR-mediated direct reprogramming

Transdifferentiation not only requires altering the expression of lineage-specific genes but also inheritable epigenetic changes. Mere overexpression of the lineage specific transcription factors is not enough to bring about efficient transdifferentiation. To overcome the epigenetic barriers microRNAs or small molecules have been used for more efficient transdifferentiation. There has been a considerable interest in utilizing targeted epigenetic tools to induce expression of lineage-specific transcription factors. Lineage-specific genes are mostly silenced in the fibroblast, and therefore require an exogenous expression of transcription factors for the reprogramming. Recently, alternatives to the overexpression of the TF transgenes have been developed, by opening the chromatin at precise loci to allow the endogenous target gene expression. Targeted epigenetic tools such as TALEN (Transcription activator-like effector nuclease) or CRISPR-based transcriptional activators such as CRISPRa-SAM (synergistic activation mediator) have been utilized to activate key genes in cellular reprogramming. One such example is activation of silenced chromatin with CRISPRa-SAM to reprogram fibroblasts into cardiac progenitor cells. Opening the silenced promoters of Gata4, Nkx2.5, and Tbx5 enabled reprogrammed fibroblasts into cardiac progenitors (Jiang et al., 2022). A simultaneous expression of endogenous lineage TF instead of overexpression of same set exogenous TF allows better reprogramming with a temporal and dosage control. Similarly, in another study, CRISPR mediated activation of neuron specific transcription factors such as Ascl1 or Ngn2 in mouse embryonic fibroblasts induced more efficient fate transition to neuronal lineage (Liu et al., 2018). Further, a dCas9 linked to the activating domain of VP64, was targeted to the promoter regions of neuronal inducers such as the BAM factors in fibroblasts to initiate the transcription (Black et al., 2016). This strategy was then further used for refining the TF involved in direct neuronal conversion (Hilton et al., 2015; Liu et al., 2018; Zhou et al., 2018). These epigenetic tools have great potential to make trans differentiation efficient and can be used in manipulating a gene of interest in case of genetic diseases.

### 6.4 Modulating epigenetic landscape using novel designed EpiBinders

A very recent epigenetic editing approach is to fuse catalytically dead Cas9 (RNA-guided endonuclease) to the core active domains of epigenetic modifier proteins. The dCas9 domain binds to the cognate sequences from guide RNA and the catalytic domain completes its function. Fusion proteins such dCas9-p300 (a Histone acetyltransferase) (Hilton et al., 2015), dCas9-DNMT3a (a DNA methyl transferase) (Stepper et al., 2017), [148] and others have been successful in altering global methylation levels. While exciting, their specificity has been a challenge since their loci-specific editing is less stringent, and it is unclear exactly why. One hypothesis is that these constructs are inevitably overexpressed in target cells, leading to an excess of catalytically active protein in the cell that then modifies DNA and chromatin (or other proteins) indiscriminately (Galonska et al., 2018). As an alternative, AI-designed small protein epigenetic binders, or EpiBinders, can adjust epigenetic Histone or DNA modifications at specific loci without increasing the functional expression level of editor proteins. The previously reported EBdCas9 (a *de novo* designed EED (a subunit of PRC2 complex)-binder, EB linked to dCas9) competes over EZH2 binding to EED and thereby inhibits histone3 lysine 27 methylation (H3K27Me3) on the specific promoter targeted by guide RNA, leading to upregulation of the specific gene expression (Levy et al., 2022). This EBdCas9/guide RNA directed tool sufficiently induced cellular reprogramming without off-target effects (Levy et al., 2022; Moody et al., 2017). One such example is EBdCas9/guide directed upregulation of a tumor suppressor p16 resulted in suppression of DMG (diffuse Midline glioma) cells (Levy et al., 2022). Similarly, designed EpiBinders to target other chromatin modifiers (DNMT, TET, histone methyltransferases, histone acetyltransferases, histone demethylases and histone deacetylases) can be utilized to activate or repress the gene of interest. After the recent development of an AI-based protein design algorithm, RFDiffusion designing small protein binders based on target structure alone has become significantly easier, enabling fast production of selective binding proteins (Watson et al., 2023). This approach now makes such efficient EpiBinder design to other epigenetic regulators highly feasible. Thus, the new generation of epigenetic editors will be smaller and modular, with the EpiBinder domains easily swapped out to match the desired modification.

### 6.5 Modulating the chromatin landscape by pioneer transcription factors

Among these various paths of cell transdifferentiation, the role of pioneer TFs is critical in rewiring the transcriptomic landscape and changing cellular identity. These factors are defined by their ability to bind closed nucleosomes and unwrap them, allowing the restructuring of chromatin for accessibility to other factors (transcription factors and epigenetic modifiers). This process of opening the chromatin is used most notably by Oct4, Sox2 and Klf4 during the reprogramming of somatic cells to the iPSC stage (Soufi et al., 2015; Chronis et al., 2017) (see Section 2 above). During the specific conversion from fibroblasts to other terminally differentiated cell types, the role of lineage related pioneer factors during the conversion process, with ASCL1 in iN conversion, MYOD in skeletal muscle and GATA4 in cardiomyocytes have been defined. Intriguingly, these factors have recently shown to have the ability to regulate the conversion toward other unrelated lineages as well; such as ASCL1, in combination with MEF2C, is shown to direct fibroblasts to a cardiomyocyte lineage (Wang et al., 2022a). To reach the full maturity however, addition of a cardiogenic specific factor, MEF2C, was required. Indeed, ASLC1 is able to bind chromatin regions corresponding to both neuronal and muscle genes, inducing chromatin opening of muscle genes to the same extent as the GMT cocktail (Wapinski et al., 2017; Wang et al., 2022a). This cross-lineage potential has been observed by MYOD which is known to maintain the muscle genome architecture but can also activate the neuronal transcriptomic landscape (Lee et al., 2020; Wang et al., 2022b). MYOD and ASCL1, both possess a very similar chromatin-binding capacity but with differences in quantitative binding (Lee et al., 2020). Blocking the muscle-specific lineage through the expression of Myt1l forced MYOD to induce the fibroblast to neuron conversion (Lee et al., 2020). This indicates the cross-lineage ability of pioneer factors drives lineage conversion through chromatin accessibility as the minimal requirement. An AI- designed transcription factor that can allow chromatin accessibility together with activation of the lineage specific genes will be one of the promising next-generation tools to regulate cell fate decision in near future. The reprogramming factor Sox2 is also able to enhance the neuronal conversion toward dopaminergic or GABA producing neurons when used in addition to other factors (Liu et al., 2012; Colasante et al., 2015; Tian et al., 2015). Recently, a triple mutant of SOX17 L111F/V118N/E122V has been reported to be more efficient than SOX2 in transactivation as well in inducing neuronal reprogramming (Weng et al., 2023). Interestingly, using the reprogramming and pioneer factors Oct4, Sox2 and Klf4 for a short time before launching the direct conversion of fibroblasts to cardiomyocytes greatly increased the transdifferentiation efficiency, by opening and priming the chromatin (Efe et al., 2011). These findings open the possibility that pioneer factors may have more general transdifferentiation potential than previously realized, and leave the door open to identifying, or AI-designing universal pioneer factors.

## 7 Conclusion

**FIGURE 3:**
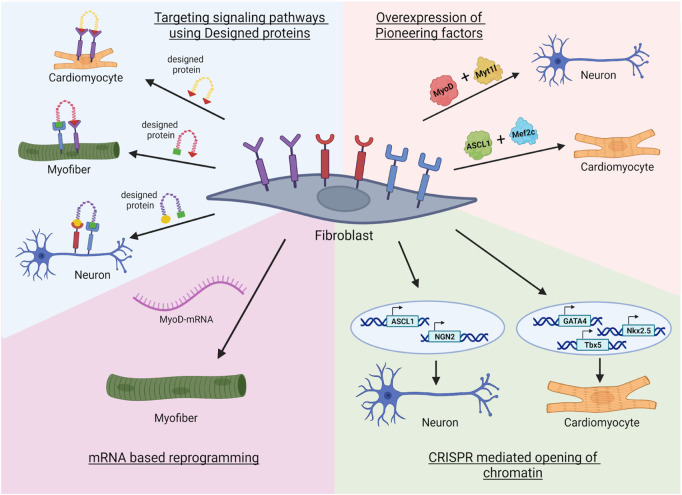
Novel approaches in transdifferentiation. First, the fibroblast receptors can be targeted with designed miniproteins that can enhance the reprogramming. Second, modified mRNA based reprogramming where transgene free expression of lineage specific transcription factors can reprogram the fibroblast to specific lineage (Qabrati et al., 2023). Third, the usage of epigenetic tools such as CRISPR mediated opening of promoters of specific pioneer transcription factor genes that are silenced in the fibroblast (Liu et al., 2018; Jiang et al., 2022). Fourth, combination of a pioneer factor with lineage specific transcription factor can open chromatin landscape allowing efficient reprogramming to multiple lineages. Image created by Biorender.

In this review, we discussed the classical methods of transdifferentiation that include transcription factors overexpression (Figure 1). Transdifferentiation has not been yet utilized at the clinical level, presumable due to low efficiency and targeting. In the future, modern tools may improve the transdifferentiation efficiency (Figure 3). Such modern tools discussed in this review are: AI-designed proteins that regulate signaling pathways or neutralize the inflammatory chemokines or cytokines, and pioneer factors or EpiBinders that open the silenced lineage genes. Further, predicted signaling pathways can also be utilized to induce a controlled transdifferentiation (Ainsworth et al., 2018; Tercan et al., 2022). These next-generation trans-differentiation approaches will come with better efficiency and plausibly with potential to treat diseases like Alzheimer’s disease, muscle injury, diabetes or myocardial infarction, resulting in elimination of the unsurmountable treatment issues at the moment (for example, finding a right donor or graft rejections). These novel approaches will enhance the efficacy and safety of direct reprogramming, allowing the ultimate decoding of the process towards plausibly resulting in 21st century personalized regenerative medicine.
